# miR-155, identified as anti-metastatic by global miRNA profiling of a metastasis model, inhibits cancer cell extravasation and colonization *in vivo* and causes significant signaling alterations

**DOI:** 10.18632/oncotarget.4942

**Published:** 2015-08-10

**Authors:** Karina G. Thomsen, Mikkel G. Terp, Rikke R. Lund, Rolf Søkilde, Daniel Elias, Martin Bak, Thomas Litman, Hans C. Beck, Maria B. Lyng, Henrik J. Ditzel

**Affiliations:** ^1^ Institute of Molecular Medicine, Department of Cancer and Inflammation Research, University of Southern Denmark, Odense, Denmark; ^2^ Department of Biomarker Discovery, Exiqon A/S, Vedbaek, Denmark; ^3^ Department of Pathology, Odense University Hospital, Odense, Denmark; ^4^ Centre for Clinical Proteomics, Department of Clinical Biochemistry and Pharmacology, Odense University Hospital, Odense, Denmark; ^5^ Department of Oncology, Odense University Hospital, Odense, Denmark

**Keywords:** miR-155, cancer, colonization, LNA miRNA microarray, *in vivo* metastasis cell line model

## Abstract

To gain insight into miRNA regulation in metastasis formation, we used a metastasis cell line model that allows investigation of extravasation and colonization of circulating cancer cells to lungs in mice. Using global miRNA profiling, 28 miRNAs were found to exhibit significantly altered expression between isogenic metastasizing and non-metastasizing cancer cells, with miR-155 being the most differentially expressed. Highly metastatic mesenchymal-like CL16 cancer cells showed very low miR-155 expression, and miR-155 overexpression in these cells lead to significantly decreased tumor burden in lungs when injected intravenously in immunodeficient mice. Our experiments addressing the underlying mechanism of the altered tumor burden revealed that miR-155-overexpressing CL16 cells were less invasive than CL16 control cells *in vitro*, while miR-155 overexpression had no effect on cancer cell proliferation or apoptosis in established lung tumors. To identify proteins regulated by miR-155 and thus delineate its function in our cell model, we compared the proteome of xenograft tumors derived from miR-155-overexpressing CL16 cells and CL16 control cells using mass spectrometry-based proteomics. >4,000 proteins were identified, of which 92 were consistently differentially expressed. Network analysis revealed that the altered proteins were associated with cellular functions such as movement, growth and survival as well as cell-to-cell signaling and interaction. Downregulation of the three metastasis-associated proteins ALDH1A1, PIR and PDCD4 in miR-155-overexpressing tumors was validated by immunohistochemistry. Our results demonstrate that miR-155 inhibits the ability of cancer cells to extravasate and/or colonize at distant organs and brings additional insight into the complexity of miR-155 regulation in metastatic seeding.

## INTRODUCTION

Metastasis is the primary cause of cancer-related deaths and remains the most significant challenge to disease management. Establishment of metastases at distant sites results from a complex cascade of events that have not yet been fully elucidated. The process involves escape of malignant cells from the primary tumor, intravasation and subsequent spread through the circulatory system (lymph or blood) to distant locations where they extravasate, colonize, induce angiogenesis and undergo expansive growth [1, 2]. While some of these disseminated cancer cells have the molecular capacity to colonize and establish metastasis, others remain dormant in the new microenvironment within distant organs. Recently, microRNAs (miRNAs), a class of small regulatory RNAs, have been implicated in metastasis development [3].

miRNAs are approximately 22 nucleotide-long, non-coding RNA molecules that regulate many different biological functions in normal cells, including growth, differentiation and apoptosis by binding to mRNA and inducing translational repression or cleavage of the mRNA target. miRNAs have been shown to be involved in both initiation and progression of cancer [4, 5]. A single miRNA can regulate multiple mRNA targets, and a single mRNA may be regulated by multiple miRNAs, therefore the specific function of a single miRNA can be difficult to elucidate.

In relation to cancer, miR-155 is a miRNA predominantly known as an oncomir that is upregulated in several cancers, including B cell lymphomas, breast, lung and colon cancers [6–10]. In addition to its oncogenic function, high miR-155 expression is also associated with lymph node metastasis and poor overall survival [8, 11, 12]. Although miR-155 is predominately known as an oncogene, it has also been found to be downregulated in human melanoma cell lines compared to healthy melanocytes, and re-expression of miR-155 led to inhibition of proliferation and induced apoptosis, suggesting a tumor suppressor role [13]. Interestingly, in triple-negative breast cancer, studies have shown that high miR-155 levels in the primary breast tumor correlate with better patient outcome, and that miR-155 inhibits metastasis development [14, 15]. These differing results highlight the need for further investigation into the role of miR-155.

Analysis of the individual steps of the complex metastatic process cannot be accomplished using patient tissue or *in vitro* assays, but *in vivo* mouse models based on inoculation of isogenic human cell lines with different phenotypes may allow studies of these processes as well as provide the means for comparative molecular screening and functional evaluation of candidate metastasis-related genes and proteins. One such metastasis model is based on the isogenic cell lines, NM-2C5 and M-4A4, which are equally tumorigenic in immunodeficient mice, but only the latter produces metastases in the lungs and lymph nodes. Although NM-2C5-derived primary tumors disseminate single cells to the lungs, they remain dormant and do not form metastases [16, 17]. Two additional cell lines, M-4A4-LM3–2 GFP (LM3) and M-4A4-LM3–4 CL-16 GFP (CL16), derived from M-4A4 by serial passage in mice, exhibit incrementally increased metastatic potential when inoculated into mice [18–20]. Hence, the model recapitulates the mechanistic steps of extravasation and colonization of circulating cancer cell at distant sites, while avoiding the inherent problems of variations in the genetic backgrounds of human tissue samples. Additionally, this model overcomes the complexities of identifying cells with metastatic potential from primary tumors [16, 17]. Protein and gene expression of NM-2C5 and M-4A4 cells have been extensively studied [21–28]. In addition, proteomic comparison of CL16 and M-4A4 has showed that the expression of only a few proteins differed between the two cell lines [26].

We describe herein a panel of 28 miRNAs that exhibited significantly altered expression in these metastatic versus non-metastatic cell lines. miR-155 exhibited the greatest alteration in expression, and further investigation of its function in these cancer cells showed that miR-155, when overexpressed in mesenchymal-like CL16 cells, inhibited their ability to extravasate, colonize and form tumors in lungs when injected into the tail vein of mice. Further, proteins exhibiting altered expression upon miR-155 upregulation were examined by comparative mass spectrometry-based proteomic analysis of xenograft tumors derived from CL16 cells overexpressing miR-155 vs. those derived from CL16 control cells. These results indicate miR-155 involvement in metastatic seeding and secondary tumor outgrowth in mesenchymal-like cancer cells.

## RESULTS

### miRNA expression profiling identified altered expression of miR-155 in metastatic vs. non-metastatic isogenic cancer cell lines

**Figure 1:**
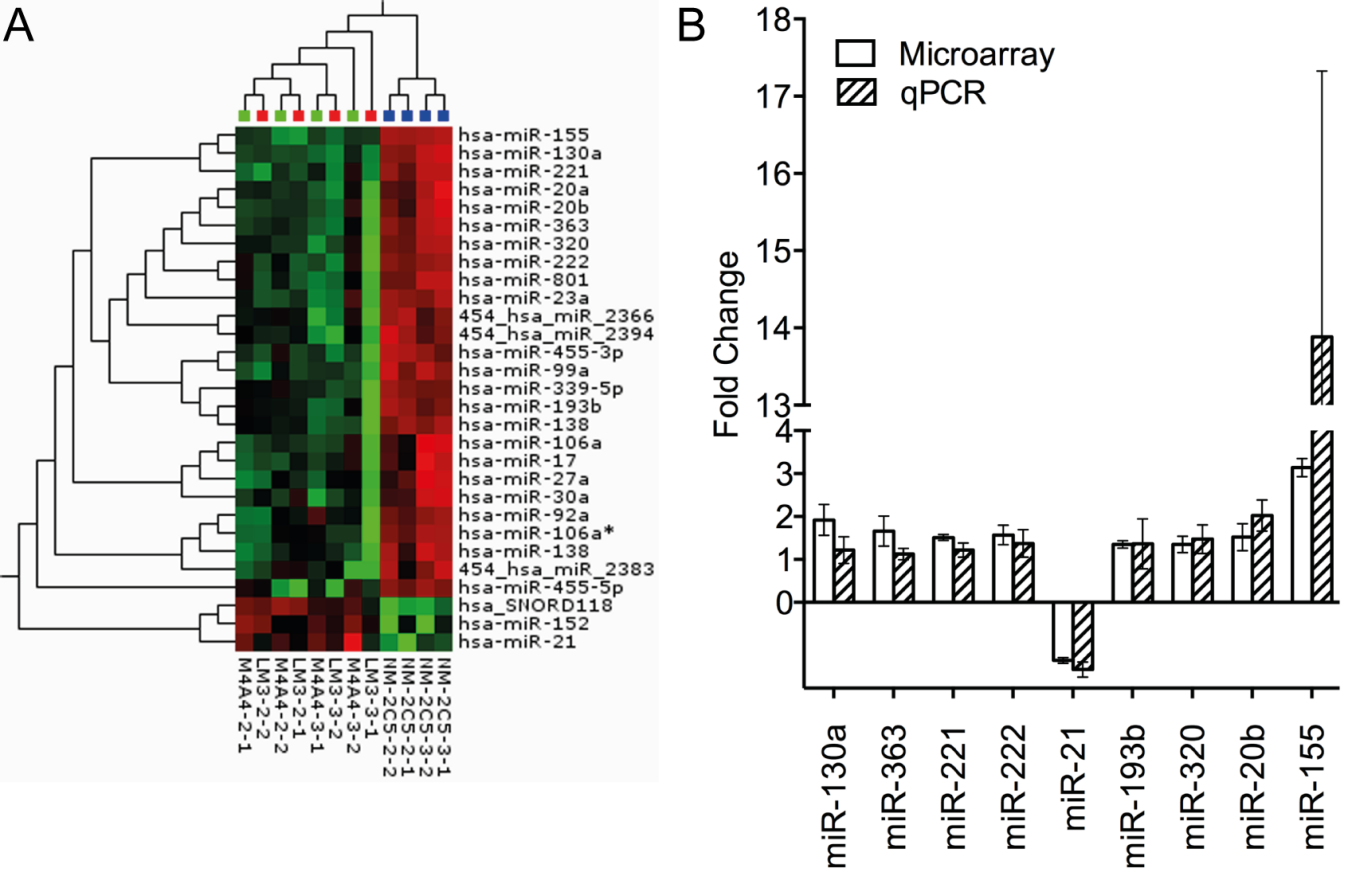
Heat map depicting unsupervised hierarchical clustering of metastatic and non-metastatic cell lines based on the differentially expressed miRNA measured by microarray and validation of miRNA alteration by qRT-PCR **A.** Top: Blue: Non-metastatic cell line NM-2C5, Green: Low metastatic cell line M-4A4, Red: Intermediate metastatic cell line LM3. Red squares in the hierarchical clustering represent higher expression and green squares lower expression of a given miRNA. **B.** Relative fold change of nine miRNAs in the non-metastatic and metastatic cell line groups measured by microarray and qRT-PCR. Values above one correspond to higher expression in the non-metastatic vs. metastatic cell line groups. Standard deviation is measured between the two biological replicates, each performed in triplicates.

Identification of miRNAs potentially associated with the ability of tumor cells to extravasate, colonize and metastasize to distant organs was accomplished by miRNA expression profiling of a metastasis model. Two biological replicates of the isogenic metastatic cancer cell lines, M-4A4 and LM3, and the non-metastatic cancer cell line NM-2C5, were compared using LNA-based microRNA microarray analysis. Each biological replicate was analyzed on two separate arrays. Raw data was deposited in the Gene Expression Omnibus (GEO) database (GSE37719). An unsupervised hierarchical clustering based on the 15 miRNAs that varied the most between samples (SD > 0.5) distinguished metastatic from non-metastatic cell lines (Suppl Fig. 1), indicating that the largest difference between all samples was their metastatic capability. Subsequently, a Student's *t*-test revealed 28 mature human miRNAs that exhibited significantly altered expression between the metastatic and non-metastatic cell line groups (Var > 0.3, *p* < 0.01 (FDR = 0.06)) (Table 1). Hierarchical clustering based on these 28 miRNAs clearly distinguished the metastatic from non-metastatic cell lines (Fig. 1A). Comparison of the miRNA profiles of the two metastatic cell lines, M-4A4 and LM3, showed no statistical differences.

**Table 1:** Twenty-eight mature miRNAs and one small nucleolar (SNORD) RNA exhibiting significantly altered expressed in isogenic non-metastatic vs. metastatic cell lines

miRNA ID	*p*-Value	FDR	Fold change
**hsa-miR-155**	4.2E-07	5.69E-05	3.14
**hsa-miR-130a**	6.4E-07	5.69E-05	1.92
**hsa-miR-363**	1.1E-04	3.31E-03	1.66
hsa-miR-138	1.1E-03	1.24E-02	1.64
hsa-miR-801	3.7E-04	8.23E-03	1.61
hsa-miR-99a	1.8E-05	1.10E-03	1.58
**hsa-miR-222**	1.7E-03	1.75E-02	1.57
**hsa-miR-20b**	1.0E-03	1.24E-02	1.52
hsa-miR-455–3p	2.7E-04	6.86E-03	1.51
**hsa-miR-221**	7.7E-05	2.77E-03	1.51
hsa-miR-23a	4.1E-04	8.24E-03	1.46
hsa-miR-20a	9.2E-04	1.24E-02	1.45
hsa-miR-106a-5p	2.9E-03	2.60E-02	1.44
454_hsa_miR_2394	7.9E-04	1.24E-02	1.44
hsa-miR-17	3.4E-03	2.77E-02	1.43
hsa-miR-106a-3p	1.8E-03	1.75E-02	1.42
hsa-miR-27a	1.1E-03	1.24E-02	1.41
hsa-miR-138	5.0E-04	8.97E-03	1.38
**hsa-miR-320**	9.6E-04	1.24E-02	1.35
hsa-miR-30a	2.3E-03	2.17E-02	1.35
**hsa-miR-193b**	3.6E-03	2.77E-02	1.35
454_hsa_miR_2366	3.5E-03	2.77E-02	1.35
hsa-miR-339–5p	6.2E-03	3.98E-02	1.34
hsa-miR-455–5p	5.4E-03	3.59E-02	1.34
hsa-miR-3648	5.1E-03	3.51E-02	1.31
hsa-miR-92a	9.6E-03	5.92E-02	1.30
hsa-miR-152	3.7E-03	2.78E-02	0.75
**hsa-miR-21**	4.5E-03	3.25E-02	0.74
hsa_SNORD118	3.0E-05	1.34E-03	0.68

Footnote: Fold change values above 1 represent miRNAs with higher expression in isogenic non-metastatic vs. metastatic cell lines. Filters: Variance > 0.3, *P* < 0.01 (FDR = 0.06). hsa denotes human miRNAs. miRNAs in bold were further analyzed using qRT-PCR. The two microRNAs, 454_hsa_miR_2394 and 454_hsa_miR_2366, were identified by 454 deep sequencing [44] and have not been annotated in miRBase. hsa-miR-801 appears to be a fragment of U11 spliceosomal RNA, and is no longer annotated as a miRNA in miRBase.

An IPA analysis focusing on the 28 mature human miRNAs exhibiting significantly altered expression between the metastatic and non-metastatic cell line groups revealed a network consisting of 16 of the 28 miRNAs associated with functions in Cancer, Gastrointestinal Disease and Hepatic System Disease (Suppl Fig. 2). miR-155, the miRNA that exhibited the highest difference in expression between metastatic and non-metastatic cell line groups (>3 fold higher in the non-metastatic), appeared to be central in this network and was deemed a top candidate for further investigation.

Nine of the 28 miRNAs exhibiting altered expression in the array analysis were selected based on the miRNA fold change, identification in the literature of cancer involvement, and whether they were central in the IPA analysis (Table 1, Suppl Fig. 2). These 9 miRNAs were examined by qRT-PCR using the miRCURY LNA™ PCR system (Fig. 1B), and all nine exhibited the same miRNA expression pattern as seen in the miRNA microarray analysis, confirming the altered miRNA expression between the non-metastatic and metastatic groups.

### High miR-155 expression in cancer cells inhibits the late steps of extravasation and/or colonization in the metastatic process

The CL16 cancer cell line, which is isogenic to NM-2C5, M-4A4 and LM3 cancer cells, but is more aggressive than M-4A4 and LM3 cells, exhibited the lowest miR-155 level of the three metastatic cell lines (Suppl Fig. 3). To examine the functional role of miR-155 expression, miR-155 was overexpressed in CL16 (CL16-miR-155) by lentiviral transduction, resulting in stable high expression of miR-155 (Suppl Fig. 3).

**Figure 2:**
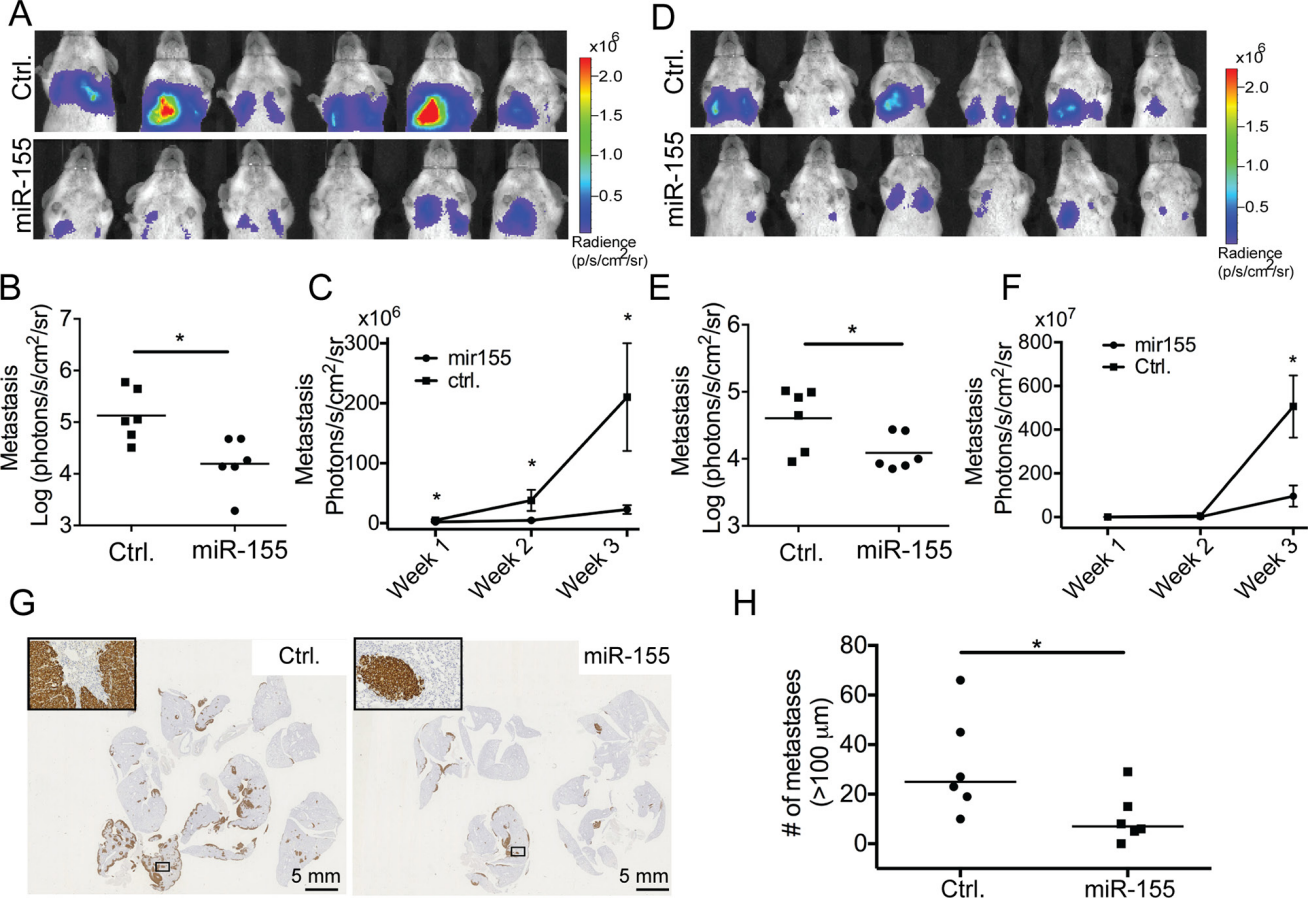
miR-155 decreases the tumor burden in lungs after i.v. injection CL16-miR-155 or CL16-Ctrl cells (7.8 × 10^5^) were injected into the tail vein of groups of female CB-17 SCID mice and tumor burden in lungs was monitored by bioluminescence imaging using an IVIS Spectrum instrument. **A.** IVIS images of the animals (photon radiance per area from the lung region) from the initial study (*n* = 6 in each group) at week 3, and **B.** comparison of the two groups using the Mann-Whitney statistical test (*p* = 0.0087). **C.** Increased tumor burden in lungs over time in the initial experiment measured by bioluminescence imaging starting one week after tumor cell injection. **D, E.** For the second animal study (*n* = 6 in each group), the IVIS scans (photon radiance per area from the lung region) at week 3 were also compared using the Mann-Whitney test (*p* = 0.041). **F.** Tumor growth in lungs in the second experiment measured by bioluminescence imaging over time starting one week after tumor cell injection. **G.** The difference in lung tumor burdens between the two groups of animals in the initial experiment was also visualized by staining the excised and FFPE lungs using an anti-human vimentin antibody. **H.** Quantitative evaluation of the differences in pulmonary foci/nodules (*n* = 6) in the initial experiment. Only tumors larger than 100 μm were included. (*p < 0.05).

To study the effect of increased miR-155 expression on the process of metastatic seeding and secondary outgrowth, 7.8 × 10^5^ CL16-miR-155 or CL16-Ctrl cells were injected intravenously (i.v.) into the tail vein of immunodeficient female CB-17 SCID mice (*n* = 6 in each group) and the tumor burden in the lungs was evaluated using bioluminescence. At endpoint, three weeks after injection, the metastatic burden was significantly lower in mice injected with CL16-miR-155 than those injected with CL16-Ctrl (*p* = 0.0087, Fig. 2A and 2B). Furthermore, measurements of the tumor burden over time showed that the CL16-miR-155 lung tumors grew slower compared to the CL16-control tumors (Fig. 2C). The observed significant difference in endpoint tumor burden after i.v. injection, as well as the difference in lung tumor growth over time were confirmed in an independent experiment (*n* = 6 in each group) using the same setup (*p* = 0.041, Fig. 2D–2F). The difference in lung tumor burdens between the two groups of animals was also assessed by staining the excised lungs with an anti-human vimentin antibody (Fig. 2G). Subsequent quantification of the stained lung tumors confirmed the significant difference in lung tumor burden at endpoint between CL16-Control and CL16-miR-155 (Fig. 2H). Together, these results indicate that miR-155 expression in CL16 cells inhibits the later steps of the metastatic process, such as extravasation and/or colonization. The expression of miR-155 in the metastases was verified using qRT-PCR at the completion of the animal study (Suppl Fig. 4).

### Evaluation of the underlying mechanism of the reduced tumor burden in lungs of high miR-155-expressing cancer cells

**Figure 3:**
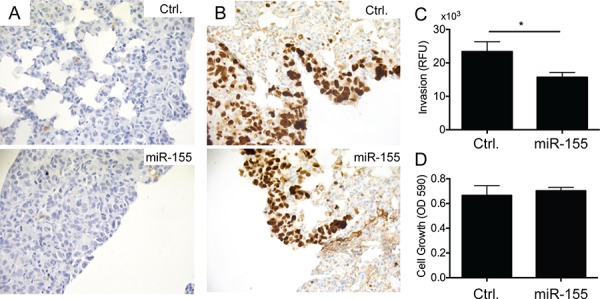
miR-155 overexpression inhibits invasion of metastatic CL16 cancer cells *in vitro*, but has no effect on proliferation or apoptosis **A.** Immunohistochemical staining of lung tumors derived from i.v. injected CL16-miR-155 or CL16-Ctrl cells for the apoptotic marker cleaved Caspase-3 showed very few apoptotic cells and no difference between the two groups. **B.** Staining of the same lung tumors for the proliferation marker Ki-67 showed similar frequency of Ki-67-positive tumors cells in the two groups. **C.** CL16-miR-155 cells showed significantly decreased invasion capability compared to CL16-Ctrl (student *t*-test, *p* = 0.028) when evaluated in an *in vitro* invasion assay. A representative experiment out of 3 is shown. **D.** In the same experiment, no difference in proliferation *in vitro* was observed between the CL16-miR-155 and CL16-Ctrl cells.

To address the underlying mechanism of the reduced tumor burden in lungs observed after injection of CL16-miR-155 cells compared to injection of CL16-Ctrl cells, we stained the corresponding lung tumors to evaluate the apoptotic marker cleaved Caspase-3 and the proliferation marker Ki-67. Very few apoptotic cells were observed, and no differences between the two groups were seen (Fig. 3A). Larger Ki-67-positive cancer cell numbers were observed in lung sections of mice injected with CL16-Ctrl cells than those injected with CL16-miR-155 cells, but as the lungs of the former group also demonstrated a larger overall tumor burden, the relative frequency of Ki-67-positive tumor cells in the two groups did not differ (Fig. 3B). We next examined CL16-miR-155 and CL16-Ctrl cells for their invasion capability using an *in vitro* invasion assay based on the Boyden chamber. CL16-miR-155 cells showed significantly decreased invasion capability compared to CL16-Ctrl cells (student *t*-test, *p* = 0.028, Fig. 3C). To assure that the difference in invasion capability was not due to a difference in proliferation of CL16-miR-155 and CL16-Ctrl cells, we concomitantly examined their proliferation *in vitro* using crystal violet and observed no difference (Fig. 3D).

### Evaluation of the influence of miR-155 on the epithelial-like/mesenchymal-like phenotype

**Figure 4:**
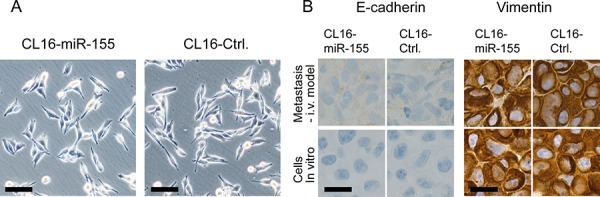
No difference in morphology, E-cadherin or vimentin expression was observed between CL16-miR-155 and CL16-Ctrl cells **A.** Phase-contrast images showing that both CL16-miR-155 and CL16-Ctrl cells exhibit mesenchymal-like morphology *in vitro*. Objective 10x, numerical aperture NA 0.3. Scale bar 100 μm. **B.** Staining for E-cadherin and vimentin in metastasis from i.v. injection model and cells grown *in vitro* as determined by immunohistochemistry. Objective 100x, numerical aperture NA 1.25 Scale bar 20 μm.

Since miR-155 has been reported to cause transition of cancer cells exhibiting an epithelial-like phenotype to a more mesenchymal-like phenotype, we evaluated possible changes in morphology and expression of E-cadherin (epithelial marker) and vimentin (mesenchymal marker) in our mesenchymal-like metastasis model. CL16-Ctrl and CL16-miR-155 cells grown *in vitro* and as xenograft tumors were analysed using contrast-phase microscopy and immunohistochemistry, respectively. Increased expression of miR-155 did not change the mesenchymal-like morphology of CL16 cells (Fig. 4A), nor did it change the expression of vimentin or E-cadherin, as both CL16-Ctrl and CL16-miR-155 cells grown *in vitro* and *in vivo* showed high expression of vimentin and no E-cadherin expression (Fig. 4B). Thus, CL16 cancer cells, which already exhibit a mesenchymal-like phenotype, retain this phenotype regardless of increased miR-155 expression.

### Identification of putative targets of miR-155

To identify proteins regulated directly or indirectly by miR-155, we compared the proteome of two CL16-miR-155-derived tumors with two CL16-Ctrl-derived tumors using mass spectrometry-based proteomics. The protein preparations of the tissue samples were separated in membrane, and soluble protein fractions and subjected to MS/MS-based proteomic analysis, which led to the identification of 3414 and 3059 proteins (4055 proteins total), respectively. Of these, 2260 and 1873, respectively, were identified by at least two unique peptides (2639 proteins total). To increase the likelihood that the proteins were indeed regulated by miR-155, we focused on those identified by at least two unique peptides, and in which at least three of the four protein ratios of miR-155/Ctrl tumors were > 1.4 fold. Using these criteria, 39 regulated proteins, 17 with lower expression and 22 with higher expression in CL16-miR-155 vs. CL16-Ctrl xenograft tumors (Suppl Table 1), were identified in the membrane protein fraction, while 56 proteins, 33 with lower and 23 with higher expression in CL16-miR-155 vs. CL16-Ctrl xenograft tumors (Suppl Table 2), were identified in the soluble protein fraction. Three proteins were identified as regulated in both the membrane and soluble samples [aldehyde dehydrogenase 1 (ALDH1A1), pirin (PIR) and acid ceramidase (ASAH1)], resulting in a total of 92 regulated proteins.

**Figure 5:**
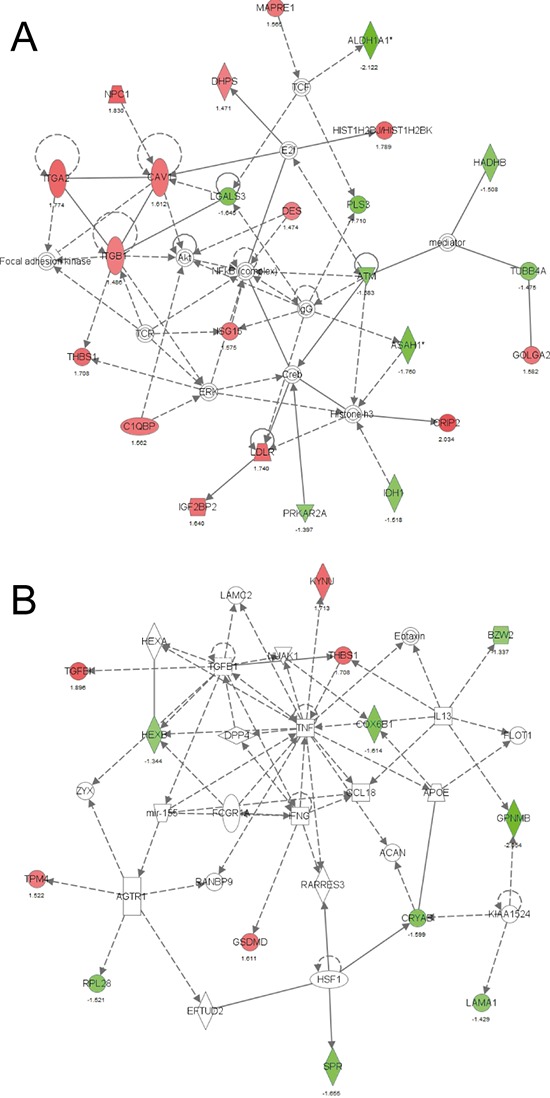
IPA network based on proteins exhibiting altered expression between CL16-miR-155- and CL16-Ctrl-derived xenograft tumors **A.** Network 1 with the highest score was related to Lipid Metabolism, Small Molecule Biochemistry, and Cellular Movement and **B.** Network 2 with the second highest score was related to Cell-To-Cell Signaling and Interaction, Cellular Growth and Proliferation, Cell Death and Survival. Red symbols indicate increased expression and green Red symbols indicate lower expression in CL16-miR-155- vs CL16-Ctrl-derived xenograft tumors.

We next investigated the interactions of these 92 regulated proteins in an IPA analysis and found two networks with high scores of 46 and 20, indicating the likelihood that the uploaded molecules in the network were linked by chance as calculated by the formula: –log(*p*-value). The main functions of the proteins in the first network, with a score of 46, were related to Lipid Metabolism, Small Molecule Biochemistry, and Cellular Movement (Fig. 5A). The network with the second highest score (*n* = 20) contained proteins with primary functions in Cell-To-Cell Signaling and Interaction, Cellular Growth and Proliferation, Cell Death and Survival (Fig. 5B). Interestingly, miR-155 was included in this second network, indicating that the proteins we identified were indeed directly or indirectly regulated by miR-155. ALDH1A1 (network 1, Fig. 5A), and PIR (the most differentially expressed protein) were selected for further validation based on involvement in IPA network, regulation level and known cancer association. The tumor suppressor protein Programmed cell death protein 4 (PDCD4) was chosen because it is a validated miR-155 target. ALDH1A1, PIR and PDCD4 were all expressed at lower levels in CL16-miR-155-derived tumors than CL16-Ctrl-derived tumors.

**Figure 6:**
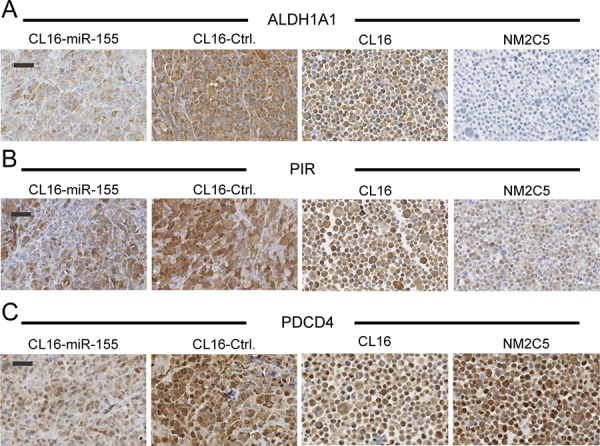
Lower expression of putative miR-155 targets in xenograft tumors with high miR-155 expression Immunohistochemical staining of ALDH1A1 **A.** PIR **B.** and PDCD4 **C.** in tumors derived from CL16 cells transduced with either miR-155 or Ctrl vector. Expression of the three targets in non-metastatic NM-2C5 (high miR-155) and metastatic CL16 cells (low miR-155) grown *in vitro* was also evaluated. Objective 40x, Scale bar 50 μm.

To validate the altered expression of ALDH1A1, PIR and PDCD4, immunohistochemical stainings were performed on FFPE tissue blocks of the xenograft tumors, and average H-scores (Suppl Table 3) were calculated (*n* = 3–6). Good agreement with regard to staining intensity of tumors generated from the same cells was observed between the different mice. ALDH1A1, PIR and PDCD4 were all found to be less intensely stained in CL16-miR-155-derived tumors than CL16-Ctrl-derived tumors (Fig. 6), confirming the proteomic results. Furthermore, immunocytochemical staining of NM-2C5 and CL16 cells showed that ALDH1A1, in contrast to CL16, was not expressed in NM-2C5 (Fig. 6A), and PIR showed weaker expression in NM-2C5 than in CL16, correlating with high miR-155 expression in NM-2C5 (Fig. 6B). For the tumor suppressor PDCD4, strong staining was observed in both NM-2C5 and CL16 (Fig. 6C).

## DISCUSSION

A high number of cancer cells dissociate from primary tumors and enter the circulation, but only a few have the ability to colonize and establish metastasis at distant sites. The metastatic process is complex and involves interactions between cancer cells and surrounding tissues in the new microenvironment, as well as cellular signaling within the cancer cells. The precise nature of these processes is poorly understood and is difficult to study in clinical samples. To gain insight into miRNA regulation in metastasis formation, we used a metastatic cell line model in which isogenic cell clones differ in their ability to extravasate, colonize, and form metastases at distant sites.

Global miRNA expression profiling identified miR-155 as the most up-regulated miRNA in the non-metastatic NM-2C5 cell line compared to the metastatic M-4A4 and LM3 cell lines. Since NM-2C5 is capable of initiating the metastatic process in disseminating single cells to distant organs, but lacks the ability to expand and establish metastasis, we posited that miR-155 may inhibit essential steps in the extravasation/colonization process. This concept was supported by our *in vivo* experiments showing that overexpression of miR-155 in highly metastatic cancer cells decreased their ability to extravasate, colonize and form tumors in lungs when injected i.v, as well as decreased their invasion ability *in vitro*. A recent study by Gasparini and colleagues [14] showed that high miR-155 expression was correlated with better patient outcome in 160 triple-negative breast cancer patients, highlighting the clinical relevance of our findings in our cancer model. Other functions of miR-155 in cancer have been reported, including a correlation between increased miR-155 levels and increased cancer invasion and migration [29]. Increased miR-155 levels have also been correlated with poor prognosis in different cancer types, including lung, liver, pancreas and breast cancer [8, 9, 11, 30]. In a recent study, stable expression of miR-155 in 4T1 breast cancer cells was found to inhibit spontaneous tumor dissemination from mammary fat pads to the lung by preventing epithelial-to-mesenchymal transition (EMT) [15]. Moreover, it was shown that miR-155 promoted tumor formation in the lung when cells were injected directly in the bloodstream. The discrepancy between their cell line models and ours likely relates to the fact that their 4T1 breast cancer cells exhibit epithelial-like characteristics, which are phenotypically influenced by miR-155, while our CL16 cell line exhibits mesenchymal-like characteristics and, in contrast, was not phenotypically influenced by miR-155.

To better understand the function of miR-155, its possible targets were identified by proteome analysis in our *in vivo* metastasis model. Network analysis of the 92 proteins exhibiting altered expression between xenograft tumors derived from high miR-155 CL16 cells compared to those from CL16-Ctrl cells revealed that the altered proteins were associated with cellular functions such as movement, growth and survival, as well as cell- to-cell signaling and interaction. Lower protein expression of three of the identified putative miR-155 targets, ALDH1A1, PIR, and PDCD4, was further confirmed by immunohistochemical staining of xenograft tumors derived from high miR-155 CL16 cells compared to those from CL16-Ctrl cells, indicating inhibition of the three proteins by miR-155. The three proteins are known to be involved in metastasis formation and could therefore be effectors of the altered miR-155 in our model. ALDH1A1 is a known cancer stem cell marker [31] associated with a poor prognosis in several cancer types [31–34]. PIR is an iron-binding protein likely functioning as a transcription cofactor, and protein expression of PIR in breast cancer tumors correlate with lymph node metastasis [35]. In melanocytic cells, knockdown of PIR has been shown to inhibit cell migration [36] and induce cellular senescence [37]. Further studies will determine whether these three proteins are direct or indirect miR-155 targets.

It should be noted that there is a continued debate as to whether the parental cell line of our cell line model, MDA-MB-435, originated from a breast cancer or a melanoma. MDA-MB-435 was originally derived from a pleural effusion of a patient with invasive ductal carcinoma [38], but the breast cancer origin was questioned when a microarray study showed a gene expression pattern primarily resembling melanoma cells [39]. However, other studies have shown that MDA-MB-435 expresses breast-specific markers and can be induced to secrete milk lipids, a characteristic unique to breast cancer cell lines, thereby confirming it is a breast cancer cell line [40]. Montel and colleagues [41] later showed that surgically-excised primary human breast cancers, as well as other human breast cancer cell lines, also expressed melanoma-related genes, demonstrating that this is a common phenomenon of breast carcinomas and confirming that MDA-MB-435 is of breast cancer origin. Whether or not MDA-MB-435 is a breast cancer cell line, the isogenic cell lines comprise a good representative model system for analyzing differences in cancer extravasation and colonization.

In summary, a panel of 28 miRNAs were identified that exhibited significantly altered expression in metastatic vs. non-metastatic cell lines and may be markers of the ability of cancer cells to colonize in distant organs. Increased expression of miR-155 in metastatic cancer cells decreased their metastatic seeding and secondary tumor outgrowth *in vivo* when injected i.v. into immunodeficient mice, further supporting a role of miR-155 in later steps of the metastatic process by inhibition of cancer cell extravasation and/or colonization in distant organs. In addition, our results and those of others indicate that the effects of miR-155 on metastasis are dependent on the epithelia-like/mesenchymal-like phenotype of the cancer cells.

## MATERIALS AND METHODS

### Cell lines and culture conditions

The human cancer cell lines NM-2C5 GFP (NM-2C5), M-4A4 GFP (M-4A4), M-4A4-LM3–2 GFP (LM3), (American Type Culture Collection (ATCC), Manassas, VA, USA) and M-4A4-LM3–4 CL-16 GFP LUC2 (CL16) [42] were cultured in Dulbecco's Modified Eagle's Medium (DMEM) (Sigma-Aldrich, MO, USA) supplemented with 10% fetal bovine serum (FBS) (Sigma-Aldrich) and 1% Penicillin/Streptomycin (Final concentration: 100 Units/ml Penicillin, 100 μg/ml Streptomycin) (Sigma-Aldrich). The isogenic cell lines NM-2C5 and M-4A4 were selected from a large panel of tumor subclones derived by serial dilution of the metastatic cancer cell line MDA-MB-435 that was systematically tested for metastatic behavior in immunodeficient mice [16]. LM3 was established by expanding isolated cells from lung metastases in mice inoculated with M-4A4 cells [18]. Similarly, cells were isolated from mice inoculated with LM3 and expanded to the M-4A4-LM3–4 CL-16 GFP (CL16) cell line [18–20]. When inoculated into immunodeficient mice, LM3 was more metastatic than M-4A4 (100% vs. 62%, respectively), establishing 2 cm tumors at 79 vs. 113 days, and 14 vs. 4 metastatic deposits per lung [18]. CL16 is even more metastatic than M-4A4 and LM3 [19, 20].

### Global miRNA array analysis

Total RNA was isolated using TRIzol (Invitrogen, Carlsbad, California, USA) from the NM-2C5, M-4A4 and LM3 cell lines according to the manufacturer's instructions. Global miRNA expression analysis was performed using the miRCURY LNA (Locked Nucleic Acid) microRNA Arrays (Exiqon, A/S, Vedbaek, Denmark) version 10.0, which, in addition to 1200 probes targeting all human, mouse and rat miRNA sequences annotated in miRBase 10.0, also include 66 probes that target proprietary microRNAs identified by deep sequencing [43, 44]. Two independent purifications were made from each cell line and miRNA expression was measured for each on two different arrays relative to one of the other cell lines using dual color array. The arrays were scanned in an Agilent G2565BA Microarray Scanner System (Agilent Technologies, Santa Clara, CA, USA), and these fluorescence images were analyzed using ImaGene™ software (BioDiscovery, CA, USA). A grid was placed on the array and the image signal and background intensities were measured for each spot in both dye channels. Normalization and background corrections were performed in the statistical programming language R with the Bioconductor [45] software package LIMMA (Linear Models for Microarray Data) [46]. Poor quality spots were removed and spots were background-corrected using the Normexp method (with an offset of 50). All fluorescence intensities were log2-transformed, and normalization was performed using LOESS (Locally weighted scatterplot smoothing) [47]. Data was visualized in heat-maps with two-way unsupervised hierarchical clustering using Qlucore Omics Explorer v.2.2 (Qlucore AB, Lund, Sweden).

### Microarray qRT-PCR verification

Verification of the miRNA microarray analysis was conducted using the miRCURY LNA™ microRNA PCR system (Exiqon) according to manufacturer's instructions. For each miRNA assay, three separate cDNA syntheses were performed for each of two biological replicates followed by a qPCR reaction. Quantitative Reverse Transcription PCR (qRT-PCR) was performed using an Applied Biosystems 7500 PCR Instrument (Applied Biosystems, Foster City, CA, USA). To verify specificity and identity of the miRNAs, a melting/dissociation curve was conducted for each miRNA after the qRT-PCR run. The baselines of the qRT-PCR runs were automatically set by the PCR instrument, while the threshold for fluorescence was manually set in the exponential phase. The inclusion criteria of Ct values were as follows: Ct value between replicates below 0.5, and amplification of at least two replicates. Furthermore, the quality of the qRT-PCR run was analyzed by examination of the amplification plot and the melting temperature curve. The stability of 5 candidate reference genes was tested using qBase v.1.3.4 [48] and geNorm [49] to identify the most stable reference genes in the three cell lines. The qRT-PCR data was normalized and analyzed using the ΔΔCt method [50]. ΔCt values were normalized using the geometric mean of the three most stable endogenous reference genes, U6 snRNA, 5S rRNA and hsa-miR-191.

### Immunocytochemistry and immunohistochemistry

Tissue and cell lines were formalin-fixed and paraffin-embedded (FFPE), and sections (4 μm) were cut from the FFPE blocks mounted on slides, dried, deparaffinized and hydrated. Endogenous peroxidase activity was blocked for 10 min with 1.5% hydrogen peroxide in TBS (Tris buffered saline) buffer, pH 7.4. Epitope retrieval for E-cadherin staining was performed by incubating slides in CC1 buffer (Ventana, Roche, Tucson, USA) for 32 min at 100°C. Slides were then incubated with mouse monoclonal anti-E-cadherin antibody (E-cadherin (36), 790–4497, Ventana, Roche) for 12 min at 36°C. Primary antibody detection was performed with OptiView (Ventana, Roche) on a Dako AutoStainerPlus (Dako). For the rest of the antibodies, a panel of epitope retrieval protocols and antibody dilutions were initially evaluated, and incubation for 15 min in TE (Tris/EDTA, pH 9) buffer (Dako) was found to be optimal for mouse monoclonal anti-vimentin antibody (VimentinV9, M0725, Dako, 1:1000). Heat-induced epitope retrieval by microwave boiling for 15 min in T-EG buffer (Tris/EGTA, pH 9) was found to be optimal for the rabbit polyclonal primary antibodies ALDH1A1 (31160002, Novus Biologicals, CO, USA, diluted 1:4000), PIR (HPA000697, Sigma-Aldrich, diluted 1:50), PDCD4 (HPA027214, Sigma-Aldrich, diluted 1:50), cleaved Caspase-3 (ASP175, Cell signaling tech, diluted 1:400) and Ki-67 (Dako M7240, diluted 1:200). Antibodies were diluted in antibody diluent S2022 (Dako, Glostrup, Denmark) and incubated on slides for 1 hour at room temperature. Slides were subsequently washed with TNT buffer (Tris/NaCl/Tween20, pH 7.5) and immunostained using EnVision™ + system-HRP labeled Polymer Anti Rabbit or Anti Mouse detection system (Dako) on a DakoAutostainerPlus (Dako). 3,3-Diaminobenzidine (DAB, Dako) was used as the substrate chromogen system for all antibodies and nuclear counterstaining was performed for 2 min in Mayer's hematoxylin. ALDH1A1, PIR and PDCD4 were evaluated using the histo-score (H-score), which combines the intensity of the staining with the number of positive cells and is calculated by adding the percentage of cells with weak intensity staining with 2 times the percentage of cells with moderate intensity staining, and then further adding 3 times the percentage of cells with strong intensity staining, resulting in a score between 0 and 300.

### Generation of CL16-miR-155 and CL16-control cells

LUC2-transduced CL16 cells [42] were harvested, washed in PBS and diluted in complete medium containing 5 μg/ml of polybrene (Sigma-Aldrich). Fifty thousand cells were transferred to a 24-well plate and transduced with miR-155 or control lentiviral particles (Applied Biological Materials Inc., Richmond, Canada) at a multiplicity of infection (MOI) of 2.5 generating stably miR-155 expressing CL16 cells (CL16-miR-155) and CL16 control cells (CL16-Ctrl).

### *In vivo* studies

Sub-confluent cells in culture were washed in PBS and harvested by scraping, resuspended in PBS at a concentration of 3.9 × 10^6^/ml, and 0.2 ml injected into the tail vein of 8-week-old female CB-17 SCID mice (Taconic). All animal experiments were approved by The Experimental Animal Committee, The Danish Ministry of Justice and performed at the animal core facility at University of Southern Denmark. The mice were housed under specific pathogen-free conditions with *ad libitum* food and drinking water. The mice were euthanized if they showed any adverse signs or symptoms of disease, including weight loss, paralysis or general discomfort. Relative quantification of tumor burden in lungs upon i.v. administration of cells was performed weekly using bioluminescent imaging (IVIS-spectrum, Perkin Elmer, Massachusetts, USA). Mice were injected with 150 mg D-luciferin/kg body weight and then anaesthetized with isoflurane gas. Images were acquired starting 10 min after luciferin injection. Regions of interest (ROI) were drawn encircling the thorax region to quantify metastases. The photon emission transmitted from the ROIs was quantified in photons/s/cm^2^/sr using Caliper Life Science Living image (version 4.2). The statistical significance of bioluminescence measurements of tumor burden in different groups was calculated using the Mann-Whitney statistical test.

### Verification of miR-155 overexpression

Verification of miR-155 expression in CL16 LUC2 cells transduced with virus packaged with vector encoding miR-155 *in vitro* and *in vivo* was conducted using the miRCURY LNA™ Universal RT microRNA PCR (Exiqon) according to manufacturer's instructions. qRT-PCR reactions were performed on a StepOnePlus™ Real-Time PCR Instrument (Applied Biosystems).

### Cell invasion assay

The cell invasion assay was performed using the QCM ECMatrix 24-well kit (Chemicon, ECM550, USA) according to the manufacturer's instructions. Each lower chamber contained 500 ul DMEM with 10%FBS as the chemoattractant. CL16-mir155 og CL16-ctrl cells (2 × 10^5^) in DMEM were placed into the upper chamber and then incubated for 48 h at 37°C in a humidified atmosphere containing 5% CO_2_. After incubation, non-migrating cells in the top chambers were completely removed by pipetting out the remaining cell suspension, and placing the invasion chamber insert into a clean well containing 225 μl of prewarmed Cell Detachment Solution for 30 minutes at 37°C. To the lower chambers 75 μl Lysis Buffer/Dye Solution was added to each well containing 225 μl cell detachment solution with the cells that invaded through the ECMatrix-coated membrane and incubation for 15 minutes at room temperature. 180 μl of the mixture was transferred of to a 96-well plate suitable for fluorescence measurement and analyzed with a fluorescence plate reader using 485/535 nm filter set. All experiments were performed in triplicate. Three independent experimental were performed with similar results.

### Target identification by proteomic analysis: Separation of soluble and membrane proteins from tumor tissue

CL16-miR-155- and CL16-Ctrl-derived xenograft tumors generated in the mammary fat pads were placed in homogenization tubes (Nalgene, Thermo Scientific, CA, USA) with 1 ml 0.1M Na_2_CO_3_, pH 11 and 600 mg 0.5 mm glass beads (Bertin Technologies, Martigny, France) and homogenized in a Precellys24 (Bertin Technologies) twice at 5000 rpm for 30 sec with incubation on ice for 2 min in between. The lysate was transferred to 4 ml ultracentrifugation tubes and 1 ml 0.1 M Na_2_CO_3_ was added to the remaining tumor tissue. This homogenization step was performed three times (6 × 5000 rpm). The lysate was incubated for 1 h at 4°C and then ultracentrifuged at 100,000 × g for 30 min at 20°C. The supernatant (containing the soluble proteins) was transferred to a second tube while approximately 300 μl 8 M Urea, 0.1 M Tris/Hcl, pH 8.5 (UA) was added to the pellet (containing the membrane proteins). The pellet was dissolved using a pestle and shaken overnight at room temperature. Five volumes of acetone were added for 1 h at −80°C to precipitate soluble proteins. The solution was centrifuged at 2300 × g for 30 min and the supernatant was removed. The pellet was washed twice with 1 ml ice-cold acetone, centrifuged at 2300 × g for 10 min and air-dried for 10 min prior to being dissolved in 500 μl UA.

### Protein desalting, digestion and iTRAQ labeling

Protein concentrations were measured in triplicate using a colorimetric assay (Bio-Rad, Hercules, CA, USA) according to the manufacturer's instructions with a BSA standard. Two hundred μg of each sample was purified using a modified version of Filter Aided Sample Preparation [51]. In brief, the samples were placed on Amicon Ultra-0.5 mL Centrifugal Filters, Ultracel-10K filters (Merck Milipore, Tullagreen, Ireland), UA was added to a final amount of 200 μl in the filter and centrifuged at 14,000 × g for 15 min. This was repeated twice followed by 15 min incubation with UA and centrifugation at 14,000 × g for 15 min (flow-through discarded). The proteins were reduced with 200 μl UA containing 10 mM DTT, placed in a closed chamber at 56°C for 30 min and centrifuged at 14,000 × g for 15 min. To alkylate the proteins, 100 μl 0.05 M iodoacetamide was added, mixed at 600 rpm for 1 min and incubated at room temperature in the dark for 20 min. The filters were then centrifuged three times at 14,000 × g for 15 min, washed twice with 100 μl 50 mM Tetraethylammonium bromide (TEAB, Sigma-Aldrich) and centrifuged at 14,000 × g for 10 min (flowthrough discarded). The proteins were then digested with Sequencing Grade Modified Trypsin (Promega, Madison, WI, USA) and mixed at 600 rpm for 1 min. Samples were incubated for 17 hours in a wet chamber at 37°C. The filters were placed in new collection tubes and centrifuged 14,000 × g for 10 min. Forty μl 50 mM TEAB was added to each filter and centrifuged 14,000 × g for 10 min. Flow-through was acidified with formic acid (FLUKA, Sigma-Aldrich) to a total concentration of 5%. In addition to the Filter Aided Sample Preparation, 500 μg of protein was directly reduced with 10 mM DTT for 30 min at 56°C and alkylated with 20 mM iodoactemide for 30 min at room temperature in the dark. These samples were digested with 10 μg Lys-C (Wako, Richmond, VA, USA) for 3 hours at room temperature, diluted 8 times with 50 mM TEAB and further digested with 10 μg Sequencing Grade Modified Trypsin (Promega). Samples from the two different digestion methods were processed in the same way. The digested peptides were desalted and concentrated on Empore C8 Extraction Disks (Empore™, Sulpeco, PA, USA), with Reverse Phase Resin Oligo R3 (Applied Biosystems) and Reverse Phase R2 (Applied Biosystems) packed in 20 μl GELoader tips (Eppendorf, Hamburg, Germany) using a modified version of that reported by Rappsilber and colleagues [52, 53]. Peptide concentrations were measured in triplicate using the colorimetric assay from Bio-Rad using a modified version of the manufacturer's instructions with a BSA standard in H_2_O. Twenty-two μg of each protein sample were iTRAQ−-labeled (iTRAQ Reagent 4Plex, AB SCIEX, MA, USA) according to manufacturer's instructions. The four soluble protein samples (iTRAQ label: 114, 115, 116 and 117) were combined in one tube, as were the four membrane protein samples. These two iTRAQ-labeled samples were then desalted and concentrated as described above.

### HILIC fractionation and nano-LC-MS/MS analysis

The iTRAQ-labeled peptides were fractionated by hydrophobic interaction liquid interaction chromatography (HILIC) using the fraction collection option on the Dionex UltiMate 3000 nano/capillary High-performance liquid chromatography (HPLC) system (Thermo Scientific). Briefly, peptide samples were loaded onto a customized HILIC column packed with TSKgel Amide-80 column material (Tosoh Bioscience LLC, PA, US) (3 μm bead size, 15 cm length, 300 μm inner diameter). The resulting fractions (*n* = 23) were analyzed in duplicate by nanoscale liquid chromatography coupled to tandem mass spectrometry (nano-LC MS/MS) using a Dionex UltiMate 3000 nano HPLC coupled to an Orbitrap Q-Exactive mass spectrometer (Thermo Scientific). Samples were separated using nano HPLC. Briefly, samples (5 μl) were loaded onto a customized fused capillary pre-column (2 cm length, 360 μm outer diameter, 75 μm inner diameter) with a flow of 5 μl per min for 7 minutes. Trapped peptides were separated on a customized fused capillary column (20 cm length, 360 μm outer diameter, 75 μm inner diameter) packed with ReproSil Pur C18 3-μm resin (Dr. Maish, Ammerbuch-Entringen, Germany) with a flow of 250 nl per min using a linear gradient from 95% solution A (0.1% formic acid) to 35% B (99.9% Acetonitrile in 0.1% formic acid) over 30 or 86 min, followed by 10 min at 90% B and 14 min at 98% A. Mass spectra were acquired in positive ion mode applying automatic data-dependent switch between one Orbitrap survey MS scan in the mass range of 400 to 1500 m/z followed by HCD (Higher-energy collisional dissociation) fragmentation and Orbitrap detection of the ten most intense ions observed in the MS scan. Target value in the Orbitrap for MS scan was 1,000,000 ions at a resolution of 70,000 at m/z 200. Peptide fragmentation in the HCD cell was performed at normalized collision energy of 30 eV. Ion selection threshold was set to 17,000 or 33,000 counts. Selected sequenced ions were dynamically excluded for 45 sec.

### Proteomic data analysis

A combined MASCOT-SEQUEST was performed where peak lists (mgf files) were processed using the Proteome Discoverer 1.4, version 1.4.0.288 (Thermo Scientific). Search parameters were set to MS accuracy 10 ppm, MS/MS accuracy 0.1 Da for HCD data, with two missed cleavages allowed, fixed modification of cysteine blocked with carbamidomethyl, and lysine and N-terminal iTRAQ, and variable modifications; methionine oxidation, lysine- and deamidated asparagines. Tandem mass spectra were searched against the Uniprot-Swissprot database, downloaded October 2012. False discovery rates were obtained using Percolator selecting identification with a *q*-value ≤ than 0.01. iTRAQ quantification was performed using Proteome Discoverer (Thermo Scientific) with reporter ion area integration within a 20 ppm window. Ratios were normalized against the median peptide ratio. The four-plex data was analyzed as ratios for each combination of the CL16-miR-155- samples (iTRAQ label 114 and 115) to the CL16-Ctrl samples (iTRAQ label 116 and 117), in all four ratios (114/116,114/117,115/116,115/117). For both the membrane and soluble protein samples, the data from Proteome Discoverer 1.4 was extracted and selected based on proteins identified by ≥ 2 unique peptides, with at least three of the four ratios above a fold-change of 1.4 and the fourth ratio at a minimum being in the same direction as the other three.

### Ingenuity Pathways Analysis

Ingenuity Pathways Analysis (IPA) (Ingenuity^®^ Systems, http://www.ingenuity.com, release date 2013–12-06) was used to evaluate whether miRNAs differentially expressed between the metastatic and non-metastatic cell lines were part of functionally related integrated and interconnected biological networks. Differentially expressed miRNA identifiers were uploaded into IPA to map and generate putative networks based on curated knowledge database of pathway interactions extracted from the literature. The gene networks were generated using both direct and indirect relationships and only molecules and/or relationships experimentally observed or highly predicted were included in the analysis. The networks were ranked by probability scores assessing miRNA inclusion in the network by chance. miRNAs that appeared to be central in relevant networks were regarded as top candidates for further investigation. An IPA analysis was also conducted for the 92 proteins regulated between the CL16-miR-155 and CL16-Ctrl xenograft tumors, including both direct and indirect relationships. Only molecules and/or relationships experimentally observed or highly predicted were included in the analysis.

### Statistical analysis

miRNAs differentially expressed between the two metastatic cell lines (M-4A4 and LM3) compared to the non-metastatic cell line (NM-2C5) in the miRNA array analysis were identified by a Student's *t*-test with Benjamini-Hochberg False Discovery Rate [54] correction (Var > 0.3, *P* < 0.01 (FDR = 0.06)). Data were visualized by heat-maps with two-way unsupervised hierarchical clustering using Qlucore Omics Explorer v.2.2 (Qlucore AB, Lund, Sweden). Statistical significance of qRT-PCR data was calculated using a Student's *t*-test (*p* < 0.05). The *p* values > 0.05 were considered non-significant.

## SUPPLEMENTARY MATERIAL FIGURES AND TABLES







## Abbreviations

miRNA: microRNA
LNA: Locked Nucleic Acid
DMEM: Dulbecco's Modified Eagle's Medium
IPA: Ingenuity Pathways Analysis
FFPE: formalin-fixed and paraffin-embedded
nano-LC MS/MS: nanoscale liquid chromatography coupled to tandem mass spectrometry

## References

[1] Chambers AF, Groom AC, MacDonald IC (2002). Dissemination and growth of cancer cells in metastatic sites. Nat Rev Cancer.

[2] Weigelt B, Peterse JL, van 't Veer LJ (2005). Breast cancer metastasis: markers and models. Nat Rev Cancer.

[3] White NM, Fatoohi E, Metias M, Jung K, Stephan C, Yousef GM (2011). Metastamirs: a stepping stone towards improved cancer management. Nat Rev Clin Oncol.

[4] Tsuchiya S, Okuno Y, Tsujimoto G (2006). MicroRNA: biogenetic and functional mechanisms and involvements in cell differentiation and cancer. J Pharmacol Sci.

[5] Iorio MV, Ferracin M, Liu CG, Veronese A, Spizzo R, Sabbioni S, Magri E, Pedriali M, Fabbri M, Campiglio M, Ménard S, Palazzo JP, Rosenberg A (2005). MicroRNA gene expression deregulation in human breast cancer. Cancer Res.

[6] Eis PS, Tam W, Sun L, Chadburn A, Li Z, Gomez MF, Lund E, Dahlberg JE (2005). Accumulation of miR-155 and BIC RNA in human B cell lymphomas. Proc Natl Acad Sci U S A.

[7] Kong W, He L, Coppola M, Guo J, Esposito NN, Coppola D, Cheng JQ (2010). MicroRNA-155 regulates cell survival, growth, and chemosensitivity by targeting FOXO3a in breast cancer. J Biol Chem.

[8] Chen J, Wang BC, Tang JH (2012). Clinical significance of microRNA-155 expression in human breast cancer. J Surg Oncol.

[9] Yanaihara N, Caplen N, Bowman E, Seike M, Kumamoto K, Yi M, Stephens RM, Okamoto A, Yokota J, Tanaka T, Calin GA, Liu CG, Croce CM (2006). Unique microRNA molecular profiles in lung cancer diagnosis and prognosis. Cancer Cell.

[10] Volinia S, Calin GA, Liu CG, Ambs S, Cimmino A, Petrocca F, Visone R, Iorio M, Roldo C, Ferracin M, Prueitt RL, Yanaihara N, Lanza G (2006). A microRNA expression signature of human solid tumors defines cancer gene targets. Proc Natl Acad Sci U S A.

[11] Greither T, Grochola LF, Udelnow A, Lautenschlager C, Wurl P, Taubert H (2010). Elevated expression of microRNAs 155, 203, 210 and 222 in pancreatic tumors is associated with poorer survival. Int J Cancer.

[12] Han ZB, Chen HY, Fan JW, Wu JY, Tang HM, Peng ZH (2012). Up-regulation of microRNA-155 promotes cancer cell invasion and predicts poor survival of hepatocellular carcinoma following liver transplantation. J Cancer Res Clin Oncol.

[13] Levati L, Alvino E, Pagani E, Arcelli D, Caporaso P, Bondanza S, Di Leva G, Ferracin M, Volinia S, Bonmassar E, Croce CM, D'Atri S (2009). Altered expression of selected microRNAs in melanoma: antiproliferative and proapoptotic activity of miRNA-155. Int J Oncol.

[14] Gasparini P, Cascione L, Fassan M, Lovat F, Guler G, Balci S, Irkkan C, Morrison C, Croce CM, Shapiro CL, Huebner K (2014). microRNA expression profiling identifies a four microRNA signature as a novel diagnostic and prognostic biomarker in triple negative breast cancers. Oncotarget.

[15] Xiang X, Zhuang X, Ju S, Zhang S, Jiang H, Mu J, Zhang L, Miller D, Grizzle W, Zhang HG (2011). miR-155 promotes macroscopic tumor formation yet inhibits tumor dissemination from mammary fat pads to the lung by preventing EMT. Oncogene.

[16] Urquidi V, Sloan D, Kawai K, Agarwal D, Woodman AC, Tarin D, Goodison S (2002). Contrasting expression of thrombospondin-1 and osteopontin correlates with absence or presence of metastatic phenotype in an isogenic model of spontaneous human breast cancer metastasis. Clin Cancer Res.

[17] Goodison S, Kawai K, Hihara J, Jiang P, Yang M, Urquidi V, Hoffman RM, Tarin D (2003). Prolonged dormancy and site-specific growth potential of cancer cells spontaneously disseminated from nonmetastatic breast tumors as revealed by labeling with green fluorescent protein. Clin Cancer Res.

[18] Goodison S, Viars C, Urquidi V (2005). Molecular cytogenetic analysis of a human breast metastasis model: identification of phenotype-specific chromosomal rearrangements. Cancer Genet Cytogenet.

[19] Montel V, Huang TY, Mose E, Pestonjamasp K, Tarin D (2005). Expression profiling of primary tumors and matched lymphatic and lung metastases in a xenogeneic breast cancer model. Am J Pathol.

[20] Suzuki M, Mose ES, Montel V, Tarin D (2006). Dormant cancer cells retrieved from metastasis-free organs regain tumorigenic and metastatic potency. Am J Pathol.

[21] Schwirzke M, Evtimova V, Burtscher H, Jarsch M, Tarin D, Weidle UH (2001). Identification of metastasis-associated genes by transcriptional profiling of a pair of metastatic versus non-metastatic human mammary carcinoma cell lines. Anticancer Res.

[22] Euer N, Schwirzke M, Evtimova V, Burtscher H, Jarsch M, Tarin D, Weidle UH (2002). Identification of genes associated with metastasis of mammary carcinoma in metastatic versus non-metastatic cell lines. Anticancer Res.

[23] Goodison S, Yuan J, Sloan D, Kim R, Li C, Popescu NC, Urquidi V (2005). The RhoGAP protein DLC-1 functions as a metastasis suppressor in breast cancer cells. Cancer Res.

[24] Lund R, Leth-Larsen R, Jensen ON, Ditzel HJ (2009). Efficient isolation and quantitative proteomic analysis of cancer cell plasma membrane proteins for identification of metastasis-associated cell surface markers. J Proteome Res.

[25] Leth-Larsen R, Lund R, Hansen HV, Laenkholm AV, Tarin D, Jensen ON, Ditzel HJ (2009). Metastasis-related plasma membrane proteins of human breast cancer cells identified by comparative quantitative mass spectrometry. Mol Cell Proteomics.

[26] Lund RR, Terp MG, Laenkholm AV, Jensen ON, Leth-Larsen R, Ditzel HJ (2012). Quantitative proteomics of primary tumors with varying metastatic capabilities using stable isotope-labeled proteins of multiple histogenic origins. Proteomics.

[27] Kreunin P, Urquidi V, Lubman DM, Goodison S (2004). Identification of metastasis-associated proteins in a human tumor metastasis model using the mass-mapping technique. Proteomics.

[28] Kreunin P, Yoo C, Urquidi V, Lubman DM, Goodison S (2007). Proteomic profiling identifies breast tumor metastasis-associated factors in an isogenic model. Proteomics.

[29] Kong W, Yang H, He L, Zhao JJ, Coppola D, Dalton WS, Cheng JQ (2008). MicroRNA-155 is regulated by the transforming growth factor beta/Smad pathway and contributes to epithelial cell plasticity by targeting RhoA. Mol Cell Biol.

[30] Han ZB, Chen HY, Fan JW, Wu JY, Tang HM, Peng ZH (2011). Up-regulation of microRNA-155 promotes cancer cell invasion and predicts poor survival of hepatocellular carcinoma following liver transplantation. J Cancer Res Clin Oncol.

[31] Ginestier C, Hur MH, Charafe-Jauffret E, Monville F, Dutcher J, Brown M, Jacquemier J, Viens P, Kleer CG, Liu S, Schott A, Hayes D, Birnbaum D (2007). ALDH1 is a marker of normal and malignant human mammary stem cells and a predictor of poor clinical outcome. Cell Stem Cell.

[32] Charafe-Jauffret E, Ginestier C, Iovino F, Tarpin C, Diebel M, Esterni B, Houvenaeghel G, Extra JM, Bertucci F, Jacquemier J, Xerri L, Dontu G, Stassi G (2010). Aldehyde dehydrogenase 1-positive cancer stem cells mediate metastasis and poor clinical outcome in inflammatory breast cancer. Clin Cancer Res.

[33] Rasheed ZA, Yang J, Wang Q, Kowalski J, Freed I, Murter C, Hong SM, Koorstra JB, Rajeshkumar NV, He X, Goggins M, Iacobuzio-Donahue C, Berman DM (2010). Prognostic significance of tumorigenic cells with mesenchymal features in pancreatic adenocarcinoma. J Natl Cancer Inst.

[34] Wang K, Chen X, Zhan Y, Jiang W, Liu X, Wang X, Wu B (2013). Increased expression of ALDH1A1 protein is associated with poor prognosis in clear cell renal cell carcinoma. Med Oncol.

[35] Shubbar E, Helou K, Kovacs A, Nemes S, Hajizadeh S, Enerbäck C, Einbeigi Z (2013). High levels of gamma-glutamyl hydrolase (GGH) are associated with poor prognosis and unfavorable clinical outcomes in invasive breast cancer. BMC Cancer.

[36] Miyazaki I, Simizu S, Okumura H, Takagi S, Osada H (2010). A small-molecule inhibitor shows that pirin regulates migration of melanoma cells. Nat Chem Biol.

[37] Licciulli S, Luise C, Scafetta G, Capra M, Giardina G, Nuciforo P, Bosari S, Viale G, Mazzarol G, Tonelli C, Lanfrancone L, Alcalay M (2011). Pirin inhibits cellular senescence in melanocytic cells. Am J Pathol.

[38] Cailleau R, Olive M, Cruciger QV (1978). Long-term human breast carcinoma cell lines of metastatic origin: preliminary characterization. In Vitro.

[39] Ross DT, Scherf U, Eisen MB, Perou CM, Rees C, Spellman P, Iyer V, Jeffrey SS, Van de Rijn M, Waltham M, Pergamenschikov A, Lee JC, Lashkari D (2000). Systematic variation in gene expression patterns in human cancer cell lines. Nat Genet.

[40] Sellappan S, Grijalva R, Zhou X, Yang W, Eli MB, Mills GB, Yu D (2004). Lineage infidelity of MDA-MB-435 cells: expression of melanocyte proteins in a breast cancer cell line. Cancer Res.

[41] Montel V, Suzuki M, Galloy C, Mose ES, Tarin D (2009). Expression of melanocyte-related genes in human breast cancer and its implications. Differentiation.

[42] Terp MG, Lund RR, Jensen ON, Leth-Larsen R, Ditzel HJ (2012). Identification of markers associated with highly aggressive metastatic phenotypes using quantitative comparative proteomics. Cancer Genomics Proteomics.

[43] Griffiths-Jones S, Saini HK, van Dongen S, Enright AJ (2008). miRBase: tools for microRNA genomics. Nucleic Acids Res.

[44] Nygaard S, Jacobsen A, Lindow M, Eriksen J, Balslev E, Flyger H, Tolstrup N, Møller S, Krogh A, Litman T (2009). Identification and analysis of miRNAs in human breast cancer and teratoma samples using deep sequencing. BMC Med Genomics.

[45] Gentleman RC, Carey VJ, Bates DM, Bolstad B, Dettling M, Dudoit S, Ellis B, Gautier L, Ge Y, Gentry J, Hornik K, Hothorn T, Huber W (2004). Bioconductor: open software development for computational biology and bioinformatics. Genome Biol.

[46] Ritchie ME, Diyagama D, Neilson J, van Laar R, Dobrovic A, Holloway A, Smyth GK (2006). Empirical array quality weights in the analysis of microarray data. BMC Bioinformatics.

[47] Smyth GK, Speed T (2003). Normalization of cDNA microarray data. Methods.

[48] Hellemans J, Mortier G, De Paepe A, Speleman F, Vandesompele J (2007). qBase relative quantification framework and software for management and automated analysis of real-time quantitative PCR data. Genome Biol.

[49] Vandesompele J, De Preter K, Pattyn F, Poppe B, Van Roy N, De Paepe A, Speleman F (2002). Accurate normalization of real-time quantitative RT-PCR data by geometric averaging of multiple internal control genes. Genome Biol.

[50] Livak KJ, Schmittgen TD (2001). Analysis of relative gene expression data using real-time quantitative PCR and the 2(-Delta Delta C(T)) Method. Methods.

[51] Wisniewski JR, Zougman A, Nagaraj N, Mann M (2009). Universal sample preparation method for proteome analysis. Nat Methods.

[52] Rappsilber J, Ishihama Y, Mann M (2003). Stop and go extraction tips for matrix-assisted laser desorption/ionization, nanoelectrospray, and LC/MS sample pretreatment in proteomics. Anal Chem.

[53] Callesen AK, Mohammed S, Bunkenborg J, Kruse TA, Cold S, Mogensen O, Christensen Rd, Vach W, Jørgensen PE, Jensen ON (2005). Serum protein profiling by miniaturized solid-phase extraction and matrix-assisted laser desorption/ionization mass spectrometry. Rapid Commun Mass Spectrom.

[54] Benjamini Y, Hochberg Y (1995). Controlling the False Discovery Rate: A Practical and Powerfull Approach to Multiple Testing. J Roy Statist.

