# Supplementary material for: miR-155, identified as anti-metastatic by global miRNA profiling of a metastasis model, inhibits cancer cell extravasation and colonization in vivo and causes significant signaling alterations
Source: Oncotarget. 2015 Aug 10;6(30):29224–39. doi: 10.18632/oncotarget.4942 (PMC4745722; doi:10.18632/oncotarget.4942)
Supplement: Supplementary file 1 [file oncotarget-06-29224-s001.pdf]

SUPPLEMENTARY FIGURES AND TABLES

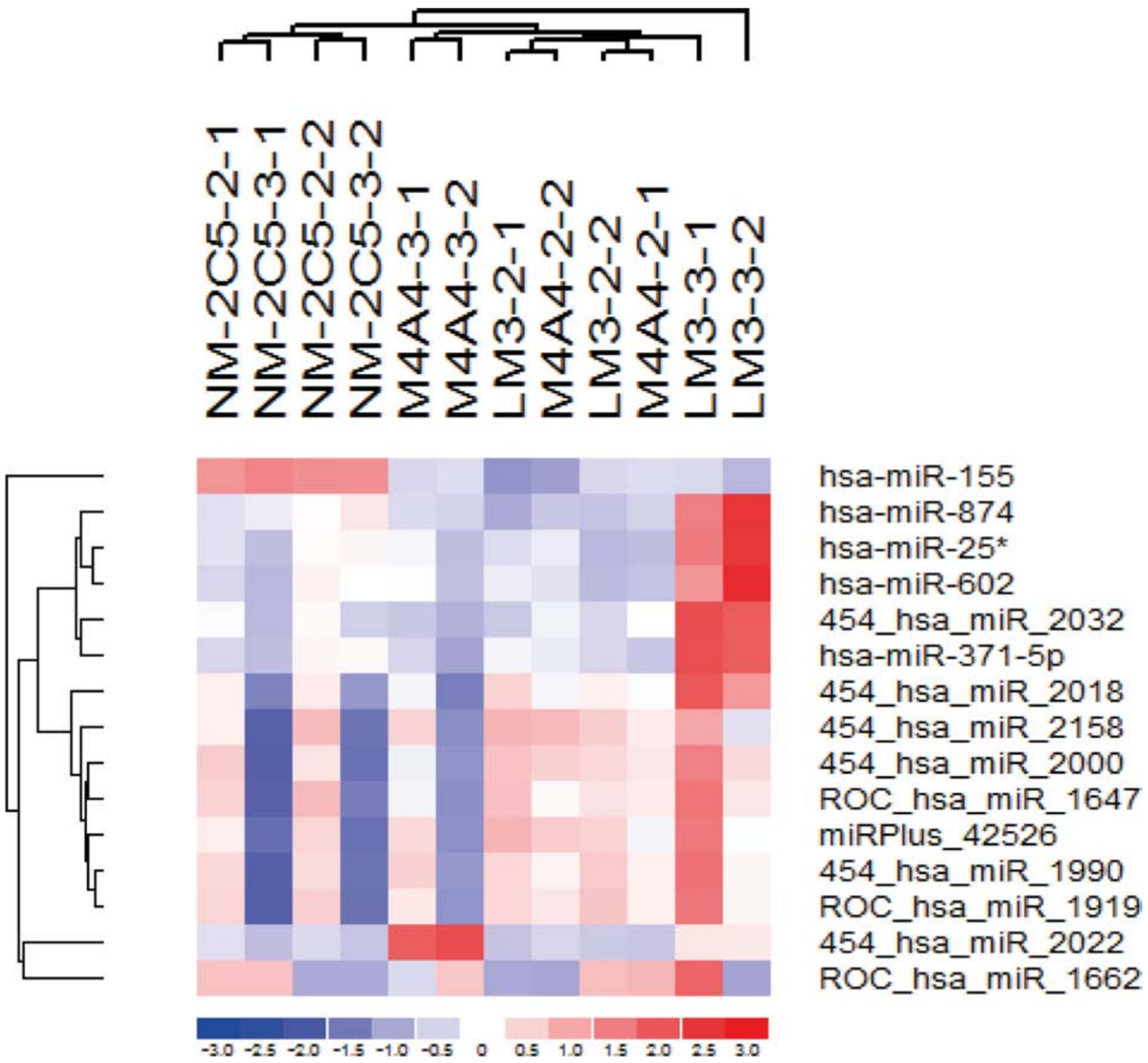

Supplementary Figure S1: Unsupervised hierarchical cluster analysis based on the 15 miRNAs that varied most between biological replicates of non-metastatic NM2C5 vs. metastatic M4A4 and LM3 cell lines. Red squares represent high expression and blue squares represent low expression of miRNA.

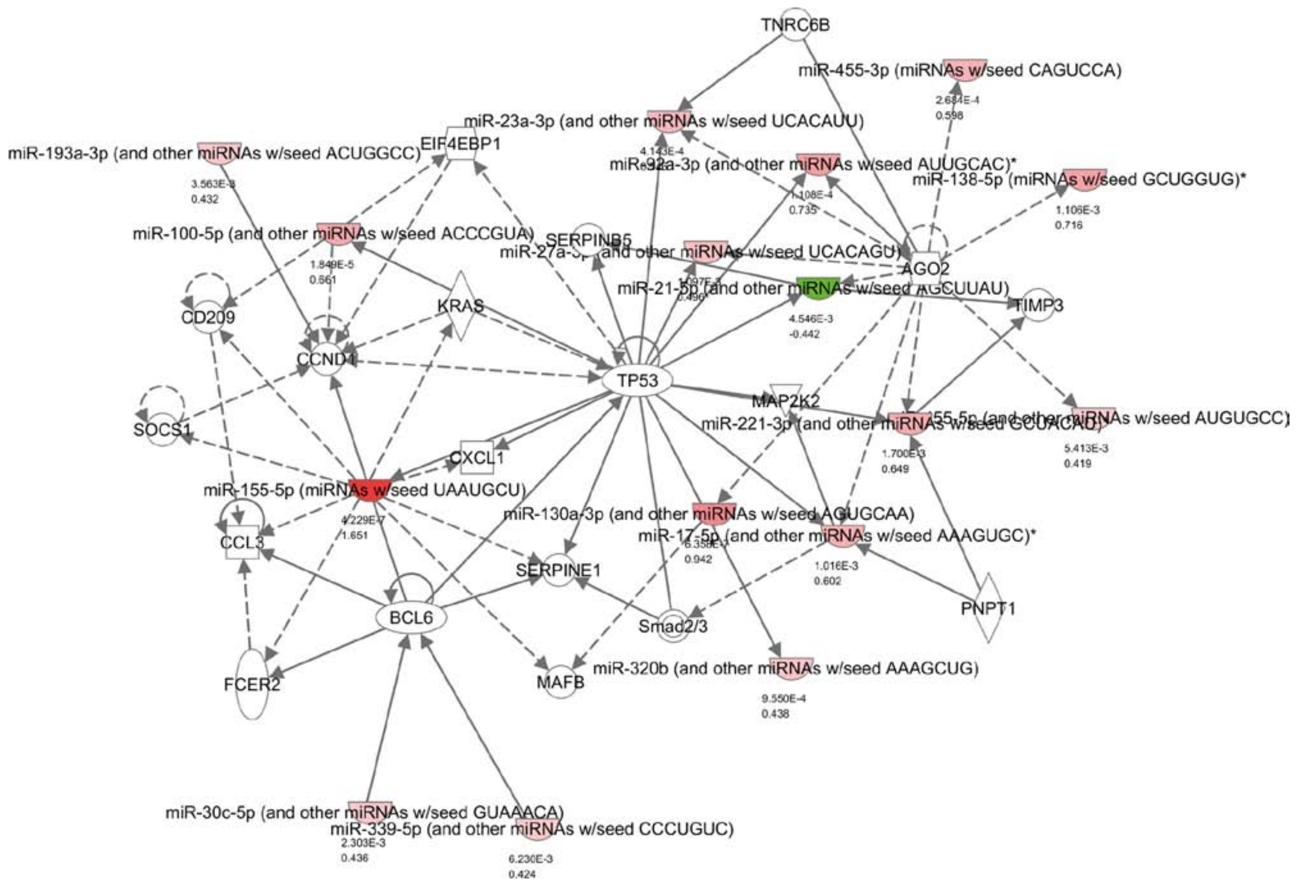

Supplementary Figure S2: Ingenuity pathway analysis (IPA) network with top functions in Cancer, Gastrointestinal Disease and Hepatic System Disease, consisting of 16 of the identified 28 miRNAs differentially expressed between the metastatic and non-metastatic cell lines. miRNAs in red are up-regulated in the non-metastatic vs. metastatic cell lines, and those in green are down-regulated.

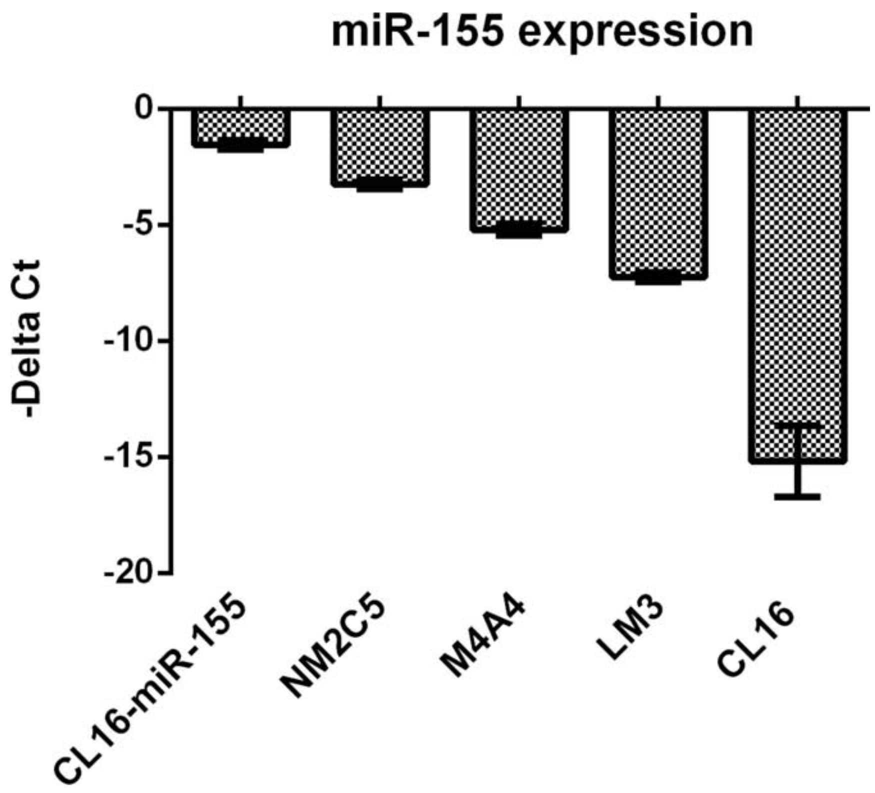

Supplementary Figure S3: Higher expression of miR-155 (lower Ct value) in the non-metastatic NM2C5 cell line and the miR-155-transduced metastatic CL16 cell line (CL16-miR-155) compared to metastatic cell lines M4A4, LM3 and CL16 using quantitative real-time PCR. SNORD44 was used a reference gene.

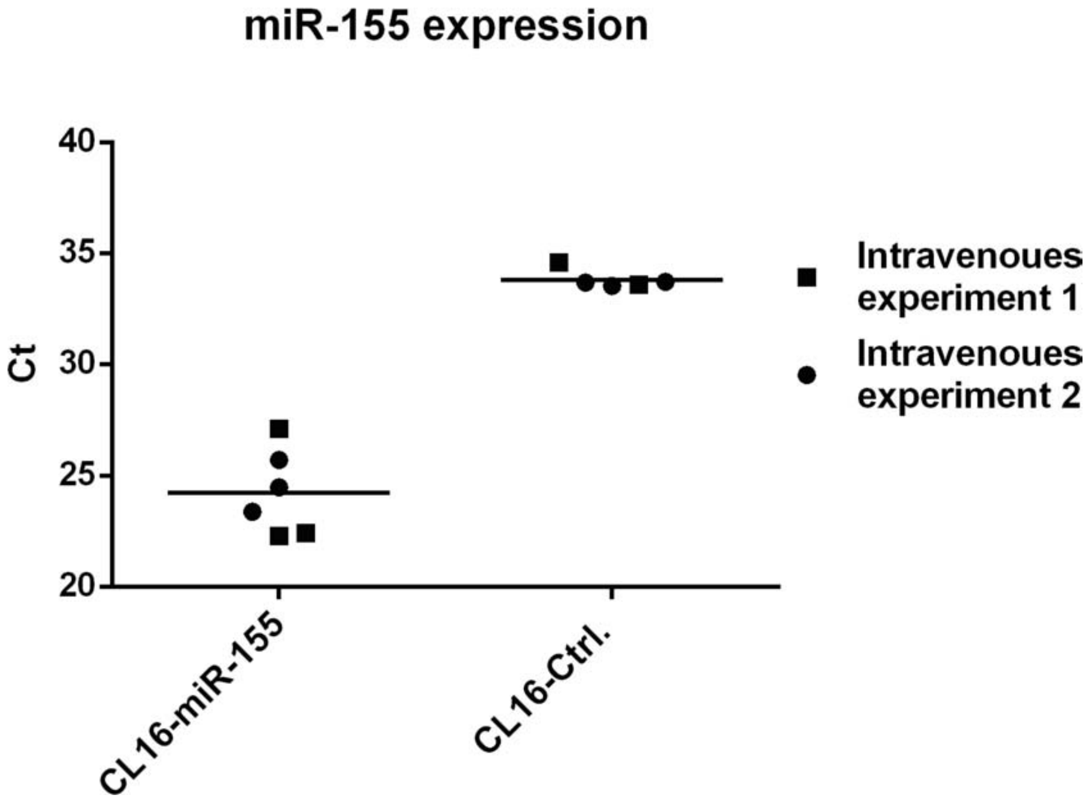

Supplementary Figure S4: miR-155 expression in randomly selected lung metastases from SCID mice injected i.v. with miR-155-transduced vs. control vector-transduced CL16 cells, showing higher (>256 fold-change) (lower Ct value) miR-155 expression in metastases of CL16-miR-155 vs. those of CL16-Ctrl cells as measured by quantitative real-time PCR.

Supplementary Table S1: In the membrane protein fraction of CL16-miR-155 vs. CL16-Ctrl derived primary tumors 39 proteins were identified as regulated, 17 with lower expression (red) and 22 with higher expression (green) in CL16-miR-155 Regulation criteria: >1.4 foldchange in at least 3 out of 4 ratios.

Supplementary Table S2: In the soluble protein fraction of CL16-miR-155 vs. CL16-Ctrl derived primary tumors 56 proteins were identified as regulated, 33 with lower (red) and 23 with higher (green) expression in CL16-miR-155. Regulation criteria: >1.4 foldchange in at least 3 out of 4 ratios.

Supplementary Table S3: Average H-score of immunohistochemical staining for ALDH1A1, PIR and PDCD4 in tumors derived from CL16 cells transduced with either miR-155 or Ctrl vector (*n* = 3−6), as well as in non-metastatic NM-2C5 and metastatic CL16 cells grown *in vitro*

H-score	ALDH1A1		PIR		PDCD4	
In vivo	CL16-miR-155	CL16-Ctl	CL16-miR-155	CL16-Ctl	CL16-miR-155	CL16-Ctl
	220	297	145	297	180	230
In vitro	NM2C5	CL16	NM2C5	CL16	NM2C5	CL16
	0	297	150	220	230	150
